# Activation of the proton-sensing GPCR, GPR65 on fibroblast-like synoviocytes contributes to inflammatory joint pain

**DOI:** 10.1073/pnas.2410653121

**Published:** 2024-12-11

**Authors:** Luke A. Pattison, Rebecca H. Rickman, Helen Hilton, Maya Dannawi, Susanne N. Wijesinghe, Graham Ladds, Li V. Yang, Simon W. Jones, Ewan St. John Smith

**Affiliations:** ^a^Department of Pharmacology, University of Cambridge, Cambridge CB2 1PD, United Kingdom; ^b^Institute of Inflammation and Ageing, University of Birmingham, Birmingham B15 2TT, United Kingdom; ^c^Department of Internal Medicine, Brody School of Medicine at East Carolina University, Greenville, NC 27834

**Keywords:** acidosis, arthritis, GPCR, inflammatory pain, nociception

## Abstract

Inflammation is associated with localized acidosis, however, attributing physiological and pathological roles to proton-sensitive receptors is challenging due to their diversity and widespread expression. Here, agonists of the proton-sensing GPCR, GPR65, were systematically characterized. The synthetic agonist BTB09089 (BTB) recapitulated many proton-induced signaling events and demonstrated selectivity for GPR65. BTB was used to show that GPR65 activation on fibroblast-like synoviocytes (FLS), cells that line synovial joints, results in the secretion of proinflammatory mediators capable of recruiting immune cells and sensitizing sensory neurons. Intra-articular injection of BTB resulted in GPR65-dependent sensitization of knee-innervating neurons and nocifensive behaviors in mice. Stimulation of GPR65 on human FLS also triggered the release of inflammatory mediators and synovial fluid samples from human osteoarthritis patients were shown to activate GPR65. These results suggest a role of GPR65 in mediating cell–cell interactions that drive inflammatory joint pain in both mice and humans.

## Significance

The lack of effective and safe drugs for pain management in chronic inflammatory conditions represents a significant unmet clinical need. Consequently, enhanced understanding of pain mechanisms is required to support novel analgesic development. The proton-sensing G-protein-coupled receptor (PS-GPCR) GPR65 is implicated in inflammatory pain, but whether the receptor coordinates nociception is unknown. Activation of GPR65 on mouse fibroblast-like synoviocytes, cells that line synovial joints, resulted in proinflammatory cytokine secretion, which increased neuronal excitability and pain-like behaviors. Furthermore, synovial fluid samples from human osteoarthritis patients activated GPR65. This work enhances understanding of PS-GPCR function, the role of cell–cell interactions in inflammatory pain and suggests that therapeutic targeting of GPR65 might represent a strategy for controlling inflammatory pain.

Chronic inflammatory conditions, such as arthritis and inflammatory bowel disease (IBD) are associated with pain, a leading complaint of those living with such diagnoses, and poorly managed by current therapeutics (1–3). As well as pain, inflammatory conditions often coincide with localized acidosis (4, 5), owing to immune cell influx (6), tissue damage (7), and regulated release of protons in response to inflammatory stimuli (8). The resulting decrease in extracellular pH exacerbates inflammation: under acidic conditions immune cells produce and secrete more proinflammatory mediators (9); additionally, protons may directly activate and sensitize sensory neurons (10, 11). Furthermore, subdermal application of acidic solutions in humans can cause pain independent of inflammation (12, 13). These findings suggest that inflammatory acidosis may contribute to the pain experienced by patients. Therefore, receptors sensitive to extracellular pH might represent points of therapeutic intervention for treating inflammatory pain.

Several receptor families are tuned to detect changes in extracellular pH, including: acid-sensing ion channels, certain transient receptor potential channels and two-pore potassium channels, and proton-sensing G-protein-coupled receptors (PS-GPCRs). While the contributions of acid-sensitive ion channels to inflammatory pain are relatively well understood, the involvement of the six PS-GPCRs is less clear (14). PS-GPCRs are expressed by several cell types involved in inflammation, including immune cells (15), fibroblasts (16), and sensory neurons (17), with increased expression reported for preclinical models of inflammation, as well as human pathologies (18–24).

Among the PS-GPCRs, GPR65 (also referred to as T Cell Death Associated Gene 8, TDAG8), shows the greatest upregulation in inflammation (18, 25). Furthermore, GPR65 knockout (KO) mice exhibit reduced pain behaviors following localized injection of acid (26), or induction of experimental arthritis (27), which has been linked to reduced infiltration of immune cells (28). Localized GPR65 knockdown also reduces the severity of mechanical hypersensitivity evoked by inflammatory insult, localized injection of acid or a GPR65 agonist (29). GPR65 has also been linked to the pathology of visceral pain conditions, including IBD (30) and cirrhosis (31). Genetic associations of *GPR65* and human pathologies including chronic obstructive pulmonary disorder (32), ankylosing spondylitis (33), ulcerative colitis (23), and atopic dermatitis (34) have also been described. Thus, understanding the signaling mechanisms of GPR65 could be of benefit across a broad range of conditions, making it an ideal candidate for elucidating the potential role of PS-GPCRs in inflammatory pain.

The widespread expression of proton-sensitive receptors makes attributing contributions of individual receptors to physiological processes and pathology a challenging task. However, in addition to protons (35), GPR65 is reported to be activated by the glycosphingolipid, psychosine (36), and the synthetic agonist BTB09089 (BTB) (37). This study first characterizes GPR65 signaling in a recombinant system, and confirms selectivity of BTB as a GPR65 agonist, before leveraging that selectivity to determine the consequences of GPR65 activation in more physiologically relevant cell types (sensory neurons and fibroblasts) and demonstrating the role of GPR65 in mediating cell–cell interactions that underpin inflammatory joint pain in mice and humans.

## Results

### BTB Recapitulates a Similar Intracellular Signaling Signature to Protons at GPR65.

**Fig. 1.**
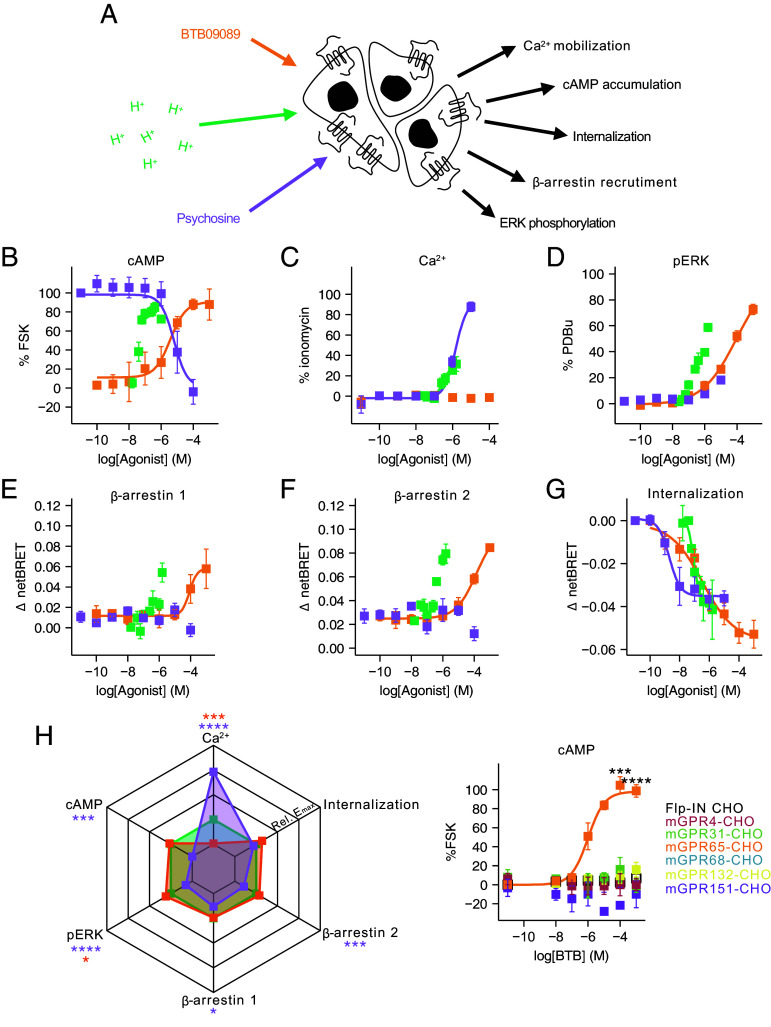
BTB recapitulates a similar intracellular signaling signature to protons at GPR65. (*A*) Intracellular signaling responses coordinated by mouse GPR65 were assessed in a CHO cell background, the ability of protons, BTB, or psychosine to coordinate (*B*) accumulation of cAMP (FSK, forskolin), (*C*) intracellular Ca^2+^ mobilization, (*D*) phosphorylation of ERK1/2 (PDBu, phorbol 12,13-dibutyrate), recruitment of (*E*) β-arrestin 1 or (*F*) β-arrestin 2 and (*G*) receptor internalization was assessed. (*H*) To compare the signaling profiles of each GPR65 agonist, the peak response of each agonist in each pathway was normalized to that achieved by proton stimulation. The E_max_ of psychosine in the cAMP pathway was set as 0. (*I*) The selectivity of BTB among other PS-GPCRs was assessed using stable cell lines and the cAMP assay. Data are from at least three independent experiments where each [agonist] was assayed in duplicate. *P-adj < 0.05, **P-adj < 0.01, ***P-adj < 0.001, ****P-adj < 0.0001: Two-way ANOVA followed by Bonferroni-corrected post hoc.

GPCRs coordinate pleiotropic intracellular signaling events, further complexity being afforded by functional selectivity, also known as biased signaling: the ability of distinct agonists to drive different responses through the same receptor. Exploring signaling bias at GPR65 among protons, BTB and psychosine could thus represent a valuable opportunity to bypass the promiscuity of proton-elicited activation in primary cells. This is because if either BTB or psychosine recapitulate the signaling signature of protons following GPR65 activation, they are more likely to be selective for GPR65 over other proton-sensitive receptors. Therefore, the ability of each agonist to evoke signaling responses was assayed in a uniform cellular background, expressing mouse GPR65 as the only known proton-sensitive receptor (mGPR65-CHO cells; Fig. 1*A*). Consistent with previous reports (35), increasing the proton concentration resulted in cAMP accumulation until pH < 6.4, when production decreased (Fig. 1*B*). Importantly proton-stimulation of parental Flp-IN cells caused no cAMP accumulation [F(1,17) = 0.139, *P =* 0.713; *SI Appendix*, Fig. S1*A*] indicating the dependence of proton-induced cAMP accumulation on GPR65 expression. Like protons, BTB also caused a concentration-dependent increase in cAMP accumulation in mGPR65-CHO cells (Fig. 1*B*). Psychosine is reported to decrease forskolin-induced cAMP accumulation (36), cells exposed to psychosine were therefore costimulated with 1 µM forskolin, and, as expected, a concentration-dependent decrease in cAMP production was observed (Fig. 1*B*). Next, the ability of protons, BTB, and psychosine to stimulate Ca^2+^ release from intracellular stores was assessed using the Ca^2+^-sensitive dye Fluo4, only psychosine, and protons evoked Ca^2+^ mobilization at concentrations above 1 µM and pH < 6.6, respectively (Fig. 1*C*). To confirm the observed increases in [Ca^2+^] were due to engagement of GPR65, experiments were repeated using parental Flp-IN cells, 10 µM psychosine having no effect (10 µM psychosine: Flp-IN, 0.33 ± 1.62%, mGPR65-CHO, 85.37 ± 6.86%, *P-adj* = 0.007; *SI Appendix*, Fig. S1*B*). Although no response was seen at pH 7, a small response was recorded when Flp-IN cells were exposed to pH 6, but this was lower than that seen in mGPR65-CHO cells (pH 6: Flip-IN, 7.58 ± 2.20%, mGPR65-CHO, 25.36 ± 0.17%, *P-adj* = 0.006; *SI Appendix*, Fig. S1*B*), indicating that protons can induce a GPR65-dependent mobilization of intracellular Ca^2+^, but that Fluo4 may have some inherent sensitivity to high concentrations of protons. Another facet of GPCR signaling is ERK activation: all three agonists coordinated ERK1/2 activation, however, efficacy differed with BTB producing the greatest response, followed by protons and last psychosine (Fig. 1*D*). Protons could not activate ERK1/2 in parental Flp-IN cells, thus highlighting the necessity of GPR65 for the observed responses in mGPR65-CHO cells (pH 6: Flp-IN, 1.11 ± 0.15%, mGPR65-CHO, 39.51 ± 2.16%, t = −17.756, df = 2.018, *P =* 0.003; *SI Appendix*, Fig. S1*C*). β-arrestins mediate GPCR desensitization of GPCRs, but can also coordinate signaling events upon recruitment to active receptors. GPR65-mediated β-arrestin recruitment was assessed using a BRET-based assay to quantify the proximity of YFP tagged β-arrestins to a luciferase tagged GPR65 (mGPR65-RLuc8). Following 15 min of exposure to protons or BTB, increased BRET was detected between GPR65-RLuc8 and β-arrestin 1-YFP (Fig. 1*E*). No change in BRET was observed upon psychosine treatment, suggesting that it does not coordinate b-arrestin recruitment to GPR65. Similar results were obtained for β-arrestin 2, with only protons and BTB able to evoke recruitment (Fig. 1*F*). Following activation many GPCRs are internalized. To assess agonist-induced GPR65 internalization, BRET assays to quantify the proximity of mGPR65-RLuc8 to a fluorescent fusion protein resident in the plasma membrane (RIT-Venus) were conducted. Following stimulation with protons, BRET between mGPR65-RLuc8 and RIT-Venus decreased in a concentration-dependent manner; BTB and psychosine also induced receptor internalization (Fig. 1*G*).

For a more comprehensive comparison of GPR65 signaling, the maximum effect (E_max_) of each agonist for each output was normalized to that achieved by proton-stimulation. This revealed that BTB has a very similar signaling fingerprint to protons, only differing in its inability to mobilize intracellular Ca^2+^ (protons, 29.88 ± 7.55%, BTB, 0.96 ± 0.79%, *P-adj <* 0.001; Fig. 1*H*) and slightly higher efficacy in the ERK pathway (protons, 58.70 ± 4.47%, BTB, 72.63 ± 6.81%, *P-adj* < 0.05; Fig. 1*H*). By contrast, psychosine coordinates a very different response profile in mGPR65-CHO cells: inhibiting of cAMP accumulation (protons, 86.42 ± 8.00%, psychosine, −4.02 ± 25.73%, *P-adj* < 0.001; Fig. 1*H*), demonstrating higher efficacy at mobilizing intracellular Ca^2+^ (protons, 29.88 ± 7.55%, psychosine, 87.66 ± 7.92%, *P-adj* < 0.0001; Fig. 1*H*), a lesser ability to activate ERK (protons, 58.70 ± 4.47%, psychosine, 18.11 ± 1.18%, *P-adj* < 0.0001; Fig. 1*H*) and an inability to recruit β-arrestins (β-arrestin 1: protons, 0.06 ± 0.02, psychosine, 0.02 ± 0.01, *P-adj* < 0.05; β-arrestin 2: protons, 0.09 ± 0.01, psychosine, 0.05 ± 0.01, *P-adj* < 0.001; Fig. 1*H*). Thus, BTB appears to represent a useful tool to mimic proton-induced signaling. A full assessment of signaling bias was not appropriate given the discrepancies in the range of concentrations tested for each agonist. Finally, the selectivity of BTB was confirmed against other PS-GPCRs using the cAMP accumulation assay. BTB only coordinated a response in cells expressing mGPR65 (mGPR65-CHO vs CHO: *P-adj* < 0.0001; Fig. 1*I*) and thus represents a selective tool for stimulating a similar intracellular signaling cascade as protons via GPR65, therefore enabling its use to study GPR65 driven physiology in cells and systems that express multiple proton-sensitive receptors.

### Intra-Articular Injection of BTB Causes Inflammation and Pain-Like Behaviors in Mice.

**Fig. 2.**
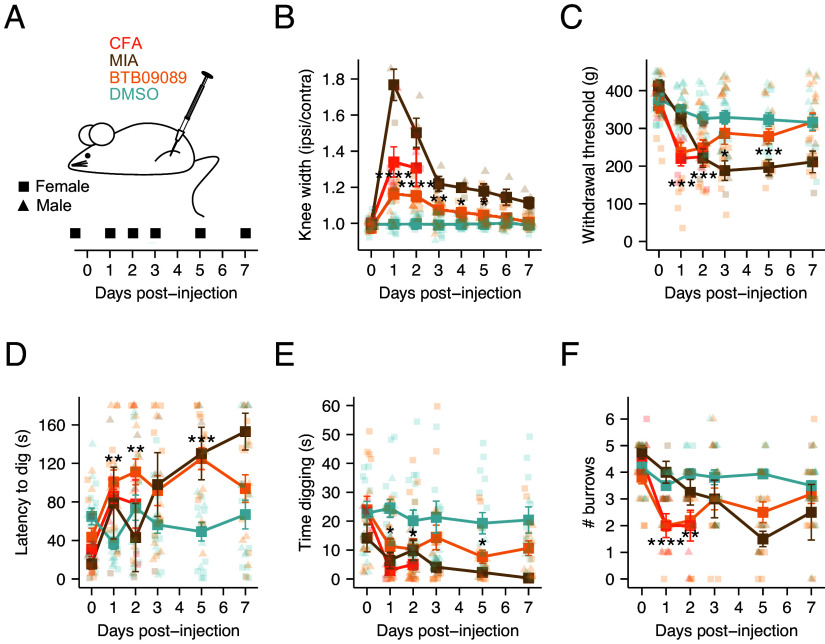
Intra-articular injection of BTB causes inflammation and pain-like behaviors in mice. (*A*) Schematic representation and experimental timeline. Mice received a unilateral injection of either 100 µM BTB, 0.1% (v/v) DMSO, 1 mg MIA or 10 µg CFA. (*B*) The ratio of the ipsilateral to contralateral knee width was calculated as a measure of the extent of inflammation. (*C*) Mechanical sensitivity of injected knee joints was determined by pressure application measurement. The (*D*) latency to dig, (*E*) time spent digging and (*F*) number of burrows dug were also measured across experimental time. *P-adj < 0.05, **P-adj < 0.01, ***P-adj < 0.001, ****P-adj < 0.0001: Repeated-measures ANOVA followed by Bonferroni-corrected post hoc; for ease of interpretation only statistical differences comparing the effect of BTB injection to the baseline measures of this group are annotated. N = 16 mice (8 female, 8 male) for BTB and DMSO groups, 6 mice (3 female, 3 male) for CFA group and 4 mice (1 female, 3 male) for the MIA group.

Having established that BTB recapitulates most features of proton-induced GPR65 signaling and is selective for GPR65 over other PS-GPCRs, the contribution of GPR65 to inflammatory joint pain was investigated. Intra-articular BTB injection served to selectively activate GPR65 in the joint environment, mimicking the increased acidity of synovial fluid common to human arthritis (38–41). After capturing baseline behaviors, mice of both sexes received a unilateral intra-articular injection of either 100 µM BTB or DMSO (0.1% v/v, vehicle control), and were studied for 7 d. Cohorts of mice injected with complete Freund’s adjuvant (CFA) or monosodium iodoacetate (MIA) were also studied as more established models of inflammatory arthritis and osteoarthritis (OA), respectively. (Fig. 2*A*). Swelling of the injected joint, as indicated by increased ipsilateral vs contralateral knee width was observed, dependent on both the substance injected and time (interaction: injection:time: F(8.89,133.42) = 25.253, *P* < 0.0001; Fig. 2*B*), comparing by substance injected revealed only BTB, CFA, and MIA resulted in joint inflammation (BTB: F(1,290) = 4.52, *P-adj <* 0.05; CFA: F(1,290) = 23.1, *P-adj* < 0.0001; MIA: F(1,290) = 21.2, *P-adj* < 0.0001; DMSO: F(1,290) = 0.001, *P-adj* = 0.973; Fig. 2*B*). Peak inflammation occurred 24-h postinjection of BTB (knee width ratio at baseline, 0.97 ± 0.02, knee width ratio at 24-h, 1.16 ± 0.02, t = −7.76, df = 15, *P-adj* < 0.0001; Fig. 2*B*), after which swelling subsided during the experimental period. Comparisons on the basis of sex revealed that female mice injected with BTB experienced greater inflammation than males [F(1,290) = 4.61, *P-adj* < 0.05], no difference on the basis of sex was seen for any other cohort. In line with knee inflammation, increased pain-like behaviors were also recorded that were dependent on the substance injected, including increased mechanical hypersensitivity of BTB-, CFA-, and MIA-injected knees (BTB: F(1,226) = 4.20, *P-adj <* 0.05; CFA: F(1,226) = 10.5, *P-adj* < 0.001; MIA: F(1,226) = 13.2, *P-adj* < 0.001; DMSO: F(1,226) = 0.032, *P-adj* = 0.859; Fig. 2*C*), an evoked pain response. The mechanical sensitivity of the contralateral joint was unaffected (interaction: injection:time: F(10,150) = 0.769, *P =* 0.621; *SI Appendix*, Fig. S2*A*). The digging behavior of mice, an ethological readout of animal pain and well-being, was also negatively affected by injection of substances causing inflammation, with increased latency to dig (BTB: F(1,226) = 6.41, *P-adj* < 0.05; CFA: F(1,226) = 2.68, *P-adj* = 0.103; MIA: F(1,226) = 18.8, *P-adj* < 0.0001; DMSO: F(1,226) = 0.101, *P-adj* = 0.751; Fig. 2*D*) and reductions in both digging duration (BTB: F(1,226) = 5.15, *P-adj* < 0.05; CFA: F(1,226) = 4.9, *P-adj* < 0.05; MIA: F(1,226) = 2.09, *P-adj* = 0.15; DMSO: F(1,226) = 0.697, *P-adj* = 0.405; Fig. 2*E*) and number of burrows produced (BTB: F(1,226) = 0.006, *P-adj =* 0.938; CFA: F(1,226) = 15.3, *P-adj* < 0.001; MIA: F(1,226) = 15.0, *P-adj* < 0.001; DMSO: F(1,226) = 1.01, *P-adj =* 0.315; Fig. 2*F*) observed. These findings likely mimic the withdrawal from everyday activities reported by human pain patients, as a result of spontaneous pain and apathy experienced as part of their inflammation (3), given mice retained full ability to use the injected joint, as inferred by the lack of an effect of injections on performance in the rotarod test (interaction: injection:time: F(10,150) = 1.666, *P* = 0.094; *SI Appendix*, Fig. S2*B*). Despite observing more severe inflammation in females in response to intra-articular injection of BTB, no sex-based differences were detected for any painlike behavior tested, although when testing mechanical sensitivity of the knee joint we found that females were generally more sensitive to mechanical pressure, for both the ipsilateral (F(1,30) = 61.69, *P <* 0.0001; Fig. 2*C*) and contralateral joint (F(1,30) = 28.341, *P* < 0.0001; *SI Appendix*, Fig. S2*A*) regardless of injection or point of testing. The swelling and painlike behaviors observed following BTB injection into the mouse knee joint align more with those seen for the CFA model of inflammatory arthritis, given they peak shortly after injection before resolving; development of painlike behaviors is seen later for the MIA model, in line with these being predominantly generated by degeneration of the joint. Overall, these data suggest that the increases in nociception and joint diameter produced by GPR65 activation are more reminiscent of an inflammatory insult to the joint. The next objective was to determine the cellular basis of GPR65-mediated inflammatory pain.

### BTB-Induced Sensitization of Sensory Neurons Depends Upon Cells Resident in the Joint.

**Fig. 3.**
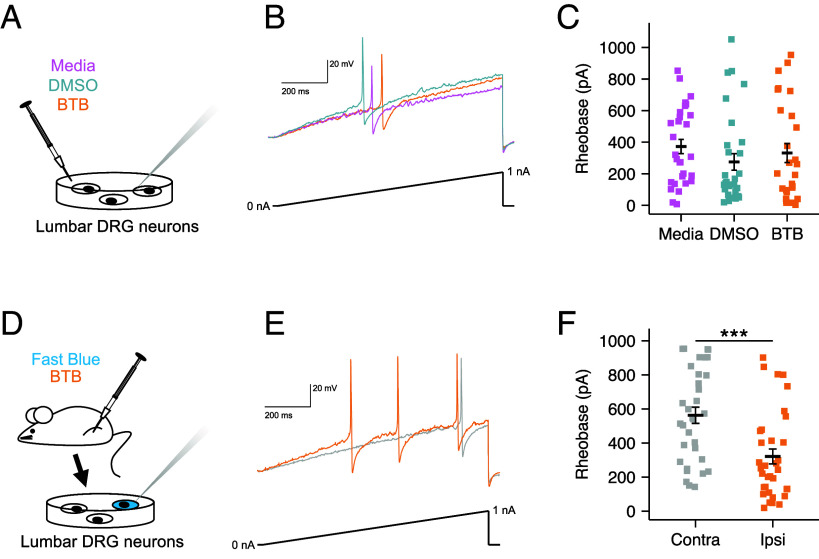
BTB-induced sensitization of sensory neurons depends upon cells resident in the joint. (*A*) Naïve lumbar (L2-L5) DRG neurons were cultured overnight with 100 µM BTB, 0.1% (v/v) DMSO or regular culture media, before electrophysiological characterization. (*B*) Representative current clamp recordings of neurons of comparable capacitance, showing action potentials evoked by ramp injection of current (0 to 1 nA, 1 s). (*C*) Stepwise current injections were used to determine the rheobase of sensory neurons. (*D*) Following retrograde labeling of knee-innervating sensory neurons (with Fast Blue), mice were injected with 100 µM BTB into one knee, 24-h postinjection DRG from the ipsilateral (Ipsi) and contralateral (Contra) were collected and cultured for electrophysiological characterization. (*E*) Representative current clamp recordings of neurons of comparable capacitance, showing action potentials evoked by ramp injection of current (0 to 1 nA, 1 s). (*F*) Stepwise current injections were used to determine the rheobase of sensory neurons. ***P < 0.001: (*C*) One-way ANOVA followed by Bonferroni-corrected post hoc, (*F*) unpaired *t*-test.

Sensory neurons, whose cell bodies reside in the dorsal root ganglia (DRG), are the principal orchestrators of nociception, transmitting noxious stimuli to the brain where they manifest as pain (42). Under inflammatory conditions sensory neurons are sensitized by mediators released by numerous cell types, giving rise to hyperalgesia, i.e. a gain in pain (43). Given the pain-like behaviors observed following BTB injection, it was hypothesized that BTB activates GPR65 on nociceptors, triggering intracellular signaling events, that sensitize these cells, leading to increased transmission of noxious stimuli and increased pain. To assess this, naïve mouse sensory neurons were dissociated from lumbar DRG and incubated with BTB, DMSO, or regular media overnight (Fig. 3*A*), akin to the greatest pain responses being seen 24-h post-intra-articular injection (Fig. 2). Electrophysiological characterization was then performed to determine cellular excitability, by investigating the amount of current required to evoke action potential discharge (rheobase; Fig. 3*B*). However, there was no difference in rheobase across the three conditions (Media, 372.41 ± 45.33 pA, DMSO, 274.67 ± 52.42 pA, BTB, 331.48 ± 59.9 pA; F(2,83) = 0.898, *P* = 0.411; Fig. 3 *B* and *C*). Similarly, no effect of any culturing condition on other intrinsic or active DRG neuron properties was measured (*SI Appendix*, Table S1).

Considering that mice injected with BTB exhibited pain-like behaviors (Fig. 2), studies were next restricted to the knee-innervating neurons, identified by retrograde tracing, of mice that had received an intra-articular BTB injection (Fig. 3*D*). As before, this cohort exhibited knee inflammation 24-h postinjection, alongside increased mechanical sensitivity and a decrease in digging behavior (*SI Appendix*, Fig. S3 *A*–*C*). Interrogation of fast blue positive, i.e. knee-innervating, neurons revealed higher excitability of cells which projected to the inflamed knee compared to those supplying the noninjected knee, as shown by their lower rheobase (Ipsi, 320.88 ± 44.00 pA vs Contra, 562.73 ± 47.62 pA, t = 3.73, df = 64.427, *P* = 0.0004; Fig. 3 *E* and *F*). A greater inward current density of macroscopic voltage-gated currents was also measured for ipsilateral neurons (Ipsi, −526.24 ± 79.14 pA/pF, Contra, −313.16 ± 41.65 pA/pF, t = 2.383, df = 17.919, *P* = 0.029; *SI Appendix*, Fig. S3 *D* and *E*). No difference was seen in any other parameter measured (*SI Appendix*, Table S2 and Fig. S3 *F*–*G*). Considered together, these results demonstrate that exposure of sensory neurons to BTB alone is insufficient to increase their excitability and suggest that other cell types within the joint environment, are necessary for BTB to induce neuronal hyperexcitability and nocifensive behaviors.

### Fibroblast-Like Synoviocytes Express GPR65 and Respond to BTB Stimulation.

**Fig. 4.**
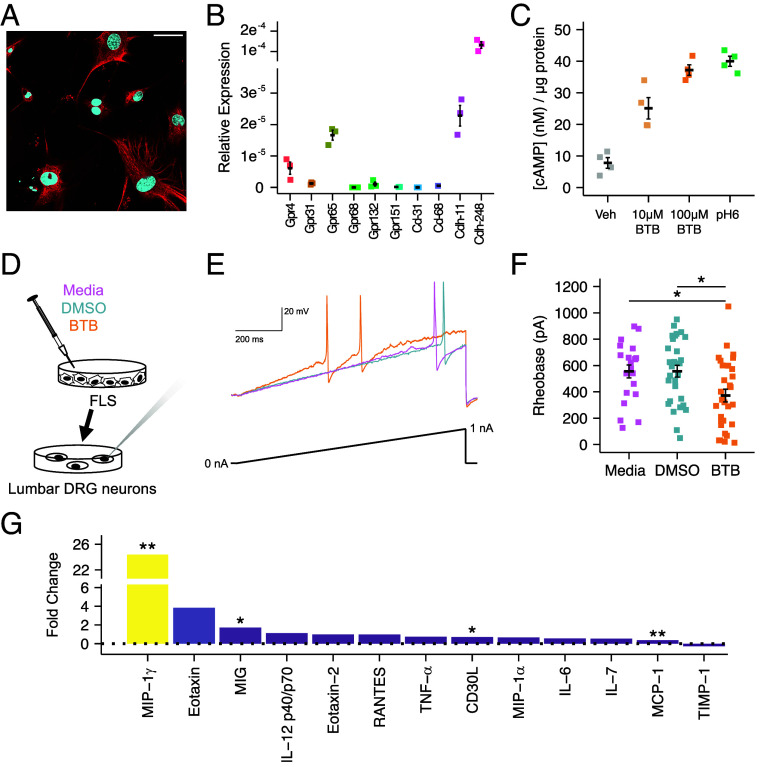
FLS express GPR65 and respond to BTB stimulation. (*A*) Mouse FLS express CDH-11 (Red: aCDH-11, Blue: Nuclear stain. (Scale bar, 50 µm.) (*B*) FLS gene expression was further interrogated via qPCR. (*C*) Intracellular FLS cAMP concentration following stimulation with pH 7.4 vehicle, BTB, or pH 6 solution. (*D*) FLS were cultured overnight with either BTB, DMSO, or regular culture media, 24-h poststimulation media were collected and later incubated with naïve DRG neurons overnight before electrophysiological characterization. (*E*) Representative current clamp recordings of neurons of comparable capacitance, showing action potentials evoked by ramp injection of current (0 to 1 nA, 1 s). (*F*) Stepwise current injections were used to determine the rheobase of sensory neurons. (*G*) BTB-induced fold change in detection of inflammatory cytokines in conditioned media from stimulated FLS. *P-adj < 0.05, **P-adj < 0.01: (*F*) One-way ANOVA followed by Bonferroni-corrected post hoc. (*G*) Two-way ANOVA followed by Bonferroni-corrected post hoc.

The involvement of multiple cell types in inflammation is becoming ever more apparent, particularly for arthritic conditions, where chondrocytes, immune cells, and osteoclasts have all been implicated in disease progression (44–46). Fibroblast-like synoviocytes (FLS), which line the synovial joints and function to secrete synovial fluid, have also been implicated in inflammatory arthritis pathology and pain (47–49). FLS also express GPR65 (50, 51) and thus the consequences of engaging GPR65 on FLS were studied. Mouse FLS cells cultured from patellae express FLS markers, such as Cdh-11 and Cdh-248, as determined by immunoreactivity and qPCR (Fig. 4 *A* and *B*), whereas expression of endothelial (Cd-31) and immune markers (Cd-68) was low (Fig. 4*B*), thus indicating high purity. qPCR analysis also revealed that among the PS-GPCRs, GPR65 was most highly expressed by FLS (Fig. 4*B*). In agreement with the expression data, stimulation of mouse FLS with either BTB or acidic pH induced cAMP accumulation (100 µM BTB, 3.72 ± 0.17 nM vs vehicle, 0.78 ± 0.17 nM, t = 9.24, df = 3, *P*-adj = 0.016; pH 6, 4.00 ± 0.16 nM vs vehicle, 0.78 ± 0.17 nM, t = 11.88, df = 3, *P*-adj = 0.008; Fig. 4*C*), consistent with the signaling responses seen in mGPR65-CHO cells (Fig. 1*A*).

To ascertain whether FLS contribute to BTB-driven neuronal hyperexcitability and pain-like behavior, FLS were stimulated with BTB or DMSO overnight before collection of media the following day. After dissociation of naïve mouse lumbar DRG, neurons were cultured overnight in the conditioned media collected from stimulated FLS prior to electrophysiological characterization (Fig. 4*D*). A lower rheobase and larger inward voltage-gated current density of neurons incubated in the media taken from BTB-stimulated FLS compared to media from either DMSO-treated FLS or unstimulated FLS cultures was observed (Rheobase: Media, 555.24 ± 49.06 pA, DMSO, 555.67 ± 44.95 pA, BTB, 372.00 ± 48.09 pA; F(2,78) = 5.22, *P* = 0.007; Fig. 4 *E* and *F*; Macroscopic inward voltage-gated current: Media, −265.84 ± 42.40 pA/pF, DMSO, −316.94 ± 46.01 pA/pF, BTB, −468.19 ± 40.55 pA/pF; F(2,27) = 6.115, *P* = 0.006; *SI Appendix*, Fig. S4 *A* and *B*), akin to the hyperexcitability previously seen in knee-innervating neurons isolated from BTB-injected mice (Fig. 3*F*). No difference was seen in any other parameter measured (*SI Appendix*, Table S3 and Fig. S4 *C* and *D*).

Taken together, these data suggest that BTB stimulation of FLS recreates the hyperexcitability of sensory neurons taken directly from BTB-injected mice. To better understand the crosstalk between BTB-activated FLS and sensory neurons, the levels of inflammatory cytokines present in media taken from BTB-stimulated FLS were examined. Compared to DMSO-stimulation, exposure of FLS to BTB resulted in increased levels of many inflammatory cytokines, most notably macrophage inflammatory protein-1 (MIP-1γ; BTB-stimulated density, 2.19 ± 0.22, DMSO-stimulated density, 0.09 ± 0.01, *P*-adj = 0.0044; Fig. 4*G*), monokine induced by interferon (MIG; BTB, 0.10 ± 0.003, DMSO, 0.04 ± 0.01, *P*-adj = 0.039; Fig. 4*G*), CD30 ligand (BTB, 0.21 ± 0.01, DMSO, 0.12 ± 0.02, *P*-adj = 0.043; Fig. 4*G*), and monocyte chemoattractant protein 1 (MCP1; BTB, 1.44 ± 0.03, DMSO, 1.05 ± 0.01, *P*-adj = 0.007; Fig. 4*G*; full analysis of cytokine levels are detailed in *SI Appendix*, Table S4). These 4 cytokines are involved in immune cell recruitment and have been associated with arthritic conditions whereby their levels correlate with inflammatory burden and concentrations of other cytokines that act on both immune cells and sensory neurons (52–55). Therefore, it is plausible that BTB stimulates FLS to trigger release cytokine release in turn recruits immune cells and sensitizes neurons.

### BTB Exerts Its Proinflammatory Effects Via GPR65 Expressing FLS.

**Fig. 5.**
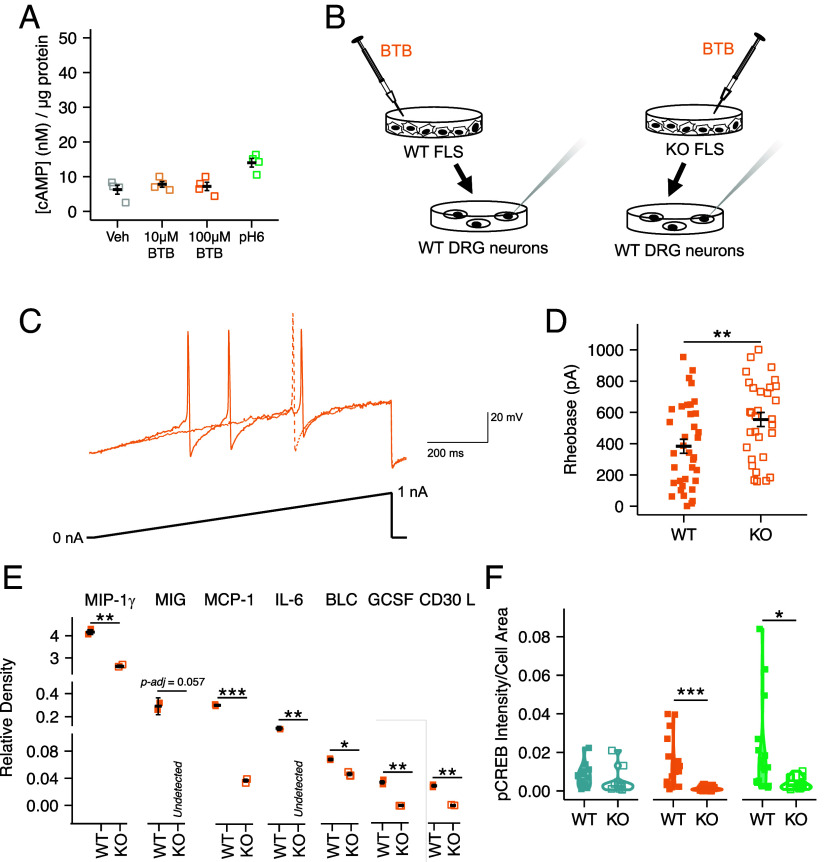
BTB exerts its pro-inflammatory effects via GPR65 expressing FLS. (*A*) Intracellular FLS cAMP concentration of FLS from GPR65 KO mice following stimulation with pH 7.4 vehicle, BTB, or pH 6 solution. (*B*) WT (closed symbols/solid lines) and KO FLS (open symbols/dashed lines) were cultured overnight with BTB, the conditioned media collected 24-h poststimulation was then incubated with naïve DRG neurons overnight before electrophysiological characterization. (*C*) Representative current clamp recordings of neurons of comparable capacitance, showing action potentials evoked by ramp injection of current (0 to 1 nA, 1 s). (*D*) Stepwise current injections were used to determine the rheobase of sensory neurons (*E*) Relative quantification of pro-inflammatory cytokines present in the conditioned media of WT and KO FLS following BTB stimulation. (*F*) phosphoCREB staining intensity of cultures of FLS from WT or KO animals following overnight stimulation with DMSO (blue), BTB (orange), or pH 6 (green). *P/*P-adj <* 0.05, **P/*P-adj* < 0.01, ***P./*P-adj* < 0.001: (*D*) unpaired *t*-test. (*E* and *F*) Two-way ANOVA followed by Bonferroni-corrected post hoc.

Results presented thus far, support the notion that BTB coordinates inflammatory joint pain in mice, most likely through its action on FLS resident in the knee joint which respond to the BTB by secreting inflammatory mediators that in turn sensitize DRG neurons. However, the requirement of GPR65 as the transducer of BTB remains to be proved. Given the lack of validated PS-GPCRs antagonists, GPR65 KO mice (56) were used to confirm the effects of BTB described with wild-type (WT) mice and cells. First, FLS were cultured from KO mice; cells displayed no cAMP accumulation when challenged with BTB (Vehicle, 628.6 ± 129.3 pM, 10 µM BTB, 781.8 ± 808.8 pM, t = 0.739, df = 3, *P*-adj = 1.00; Fig. 5*A*) and an attenuated pH 6 response compared to that seen for WT FLS (Vehicle, 628.6 ± 129.3 pM, pH 6, 1.41 ± 0.12 nM, t = 12.385, *P*-adj = 0.007; pH 6 WT, 4.00 ± 0.16 nM, pH 6 KO, 1.41 ± 0.12 nM, t = −10.58, df = 3, *P*-adj = 0.002; Figs. 4*C* and 5*A*). To determine whether GPR65 is necessary for the release of inflammatory mediators shown to sensitize DRG neurons, FLS cultured from WT or KO mice were stimulated with BTB overnight, conditioned media collected the following day was then incubated with naïve WT DRG neurons overnight before electrophysiological characterization (Fig. 5*B*). The rheobase of DRG neurons cultured in the media from WT BTB-stimulated-FLS was markedly reduced compared to neurons cultured in the media from KO FLS (WT, 383.61 ± 44.55 pA, KO, 554.52 ± 44.98 pA, t = 2.670, df = 64.515, *P* = 0.0089; Fig. 5 *C* and *D*). Neurons incubated with the media from WT FLS stimulated with BTB also exhibited larger voltage-gated inward current densities (WT, −539.00 ± 56.47 pA/pF, KO, −315.63 ± 49.97 pA/pF, t = 2.963, df = 19.708, *P* = 0.008; *SI Appendix*, Fig. S5 *A* and *B*). Whether DRG neurons were cultured in conditioned media from WT or KO FLS did not affect any other intrinsic or active property (*SI Appendix*, Table S5 and Fig. S5 *C* and *D*).

Among the cytokines released in response to BTB-stimulation of WT FLS (Fig. 4*G*), reduced levels were found in the media of KO FLS following exposure to BTB (MIP-1: WT, 4.16 ± 0.10, KO, 2.62 ± 0.05, *P*-adj = 0.0058; MIG: WT, 0.29 ± 0.07, KO, undetected, *P*-adj = 0.0569; CD30 ligand: WT, 0.03 ± 0.002, KO, undetected, *P*-adj = 0.0037; MCP1: WT, 0.30 ± 0.01, KO, 0.04 ± 0.002, *P*-adj = 0.000713; Fig. 5*E*). The comparison of BTB-stimulation of WT vs KO FLS also revealed that BTB stimulation of GPR65 resulted in higher levels of other proinflammatory cytokines such as granulocyte colony-stimulating factor (GCSF; WT, 0.03 ± 0.002, KO, undetected, *P*-adj = 0.0041; Fig. 5*E*), interleukin 6 (IL-6; WT, 0.12 ± 0.01, KO, undetected, *P*-adj = 0.0094; Fig. 5*E*) and B lymphocyte chemoattractant (BLC; WT, 0.07 ± 0.002, KO, 0.05 ± 0.002, *P*-adj = 0.0156; Fig. 5*E*), among others; full analysis detailed in *SI Appendix*, Table S6. GCSF, IL-6 and BLC all have established roles in immune cell recruitment and joint degeneration in arthritis (57–59), IL-6 is additionally thought to directly sensitize sensory neurons to induce mechanical and thermal hypersensitivity (60, 61). These findings suggest that BTB activation of GPR65 on FLS might coordinate the expression and secretion of proinflammatory cytokines. To further explore the role of GPR65 in regulation of gene expression further, the ability of BTB to induce phosphorylation, and thus activation, of CREB, a transcription factor downstream of the Gas/cAMP pathway was examined in FLS via immunostaining of phopsho-CREB (pCREB). Compared to KO FLS, higher pCREB intensity was observed when WT FLS were stimulated with BTB (WT, 0.015 ± 0.003, KO, 0.001 ± 0.000, *P*-adj = 0.00051; Fig. 5*F*). Importantly DMSO treatment had no effect on pCREB staining in FLS of either genotype (WT, 0.007 ± 0.001, KO, 0.005 ± 0.002, *P*-adj = 0.246; Fig. 5*F*). To begin to address whether GPR65 might influence the FLS transcriptome under more endogenous conditions, the effect of pH 6 treatment on FLS was also examined and resulted in higher pCREB intensity staining for WT vs KO FLS (WT, 0.020 ± 0.005, KO, 0.004 ± 0.001, *P*-adj = 0.0198; Fig. 5*F*). This suggests that the acidity of synovial fluid in arthritic conditions might modulate FLS via GPR65 to induce transcription machinery that results in the secretion of proinflammatory mediators and contribute to arthritic pathology.

### GPR65 KO Mice Do Not Develop Joint Inflammation or Pain Following Intra-Articular Injection of BTB.

**Fig. 6.**
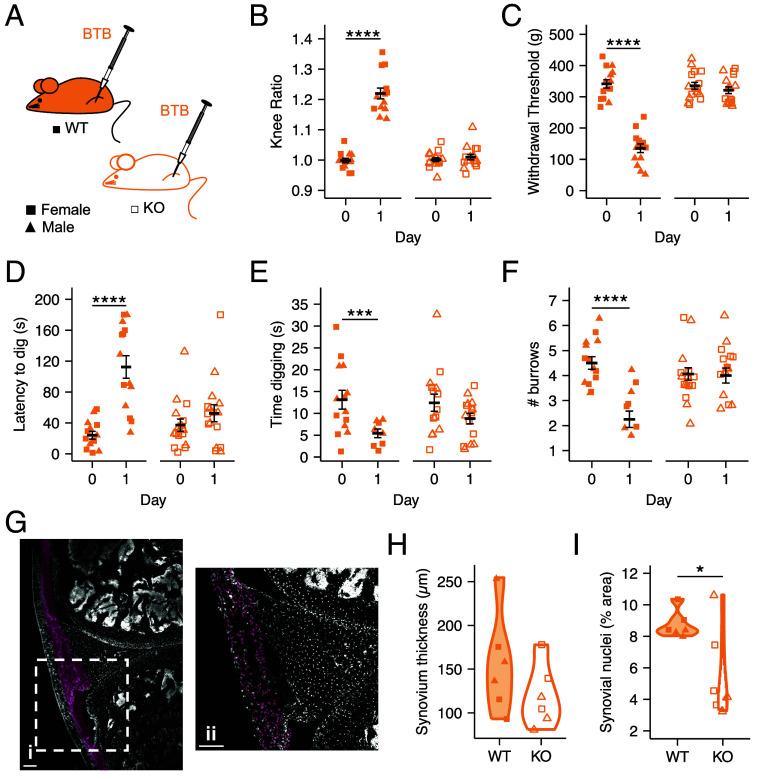
GPR65 KO mice do not develop joint inflammation or pain following intra-articular injection of BTB. (*A*) WT (WT, closed symbols) and GPR65 KO, open symbols) mice of either sex (females, square symbols; males, triangular symbols) received unilateral intra-articular injections of 100 µM BTB. (*B*) The ratio of the ipsilateral to contralateral knee width was calculated as a measure of the extent of inflammation. (*C*) Mechanical sensitivity of the injected knee joints was determined by pressure application measurement. The (*D*) latency to dig, (*E*) time spent digging and (*F*) number of burrows dug were also measured across experimental time. Following conclusion of the behavioral study, ipsilateral knee joints were collected and examined for histological changes. (*G*) Representative nuclei staining of an ipsilateral knee collected from a KO animal: i pink area represents the synovium, ii pink puncta represent automatically detected nuclei within the synovial perimeter (Scale bars, 100 µm.) The (*H*) synovium thickness and (*I*) percentage of synovial area occupied by nuclei was compared between WT and KO mice. *P < 0.05, ***P-adj < 0.001, ****P-adj < 0.0001: (*B*–*F*) repeated measures ANOVA followed by Bonferroni-corrected post hoc. (*H* and *I*) unpaired *t*-test. (*B*–*F*): N = 14 WT mice (7 female, 7 male) and 16 KO mice (8 female, 8 male). (*H* and *I*): N = 6 WT mice (3 female, 3 male) and 6 KO mice (3 female, 3 male).

To confirm that GPR65 is responsible for the pain-like behaviors observed following intra-articular BTB knee injection (Fig. 2), cohorts of WT and KO mice were injected with BTB into the knee joint; joint swelling and pain-like behaviors were measured at baseline and 24-h postinjection, coinciding with the peak of BTB-induced inflammation (Fig. 2*B*). As before, intra-articular BTB knee injection caused swelling in WT mice (knee width ratio: Baseline, 1.00 ± 0.01, 24-h, 1.22 ± 0.02, t = −10.72, df = 13, *P*-adj < 0.0001; Fig. 6*B*), but no inflammatory response was evoked in KO mice (knee width ratio: Baseline, 1.00 ± 0.01, 24-h, 1.01 ± 0.01, t = −0.982, df = 15, *P*-adj = 0.342; Fig. 6*B*). In agreement with earlier results (Fig. 2*B*) WT female mice experienced greater inflammation than males (knee width ratio at 24-h: Female, 1.26 ± 0.03, Male, 1.18 ± 0.01, t = 3.92, df = 6, *P*-adj = 0.008; Fig. 6*B*). The increased mechanical sensitivity of ipsilateral joints of WT mice (Baseline, 341.33 ± 13.16 g, 24-h, 135.38 ± 13.87 g, t = 10.04, df = 13, *P*-adj < 0.0001; Fig. 6*C*) was not observed for KO mice (Baseline, 335.18 ± 11.18 g, 24-h, 320.94 ± 10.81 g, t = 0.839, df = 15, *P*-adj = 0.415; Fig. 6*C*). Neither genotype showed any change in mechanical sensitivity of the contralateral joint (F(1,52) = 0.041, *P* = 0.84; *SI Appendix*, Fig. S5). Digging behaviors were also unaffected following BTB administration in KO mice (latency: Baseline, 37.13 ± 8.00 s, 24-h, 52.44 ± 11.06 s, t = −1.09, df = 15, *P*-adj = 0.294; Fig. 6*D*; duration: Baseline, 11.68 ± 1.99 s, 24-h, 8.26 ± 1.26 s, t = 1.79, df = 15, *P*-adj = 0.094; Fig. 6*E*; burrows: Baseline, 4.06 ± 0.25, 24-h, 4.00 ± 0.30, t = 0.169, df = 15, *P*-adj = 0.868; Fig. 6*F*). Deficits in digging were however seen 24-h postinjection of WT mice (latency: Baseline, 24.00 ± 5.03 s, 24-h, 112.43 ± 14.70 s, t = −7.55, df = 13, *P*-adj < 0.0001; Fig. 6*D*; duration: Baseline, 13.13 ± 2.14 s, 24-h, 3.17 ± 0.91 s, t = 4.88, df = 13, *P*-adj = 0.0003; Fig. 6*E*; burrows: Baseline, 4.5 ± 0.25, 24-h, 1.93 ± 0.36, t = 9.47, df = 13, *P*-adj < 0.0001; Fig. 6*F*). Despite the inflammatory response following injection of BTB to WT differing for females and males, no sex effect was seen for any behavioral readout (Mechanical threshold: F(1,52) = 2.84, *P*-adj = 0.098; Digging latency: F(1,52) = 0.132, *P*-adj = 0.718; Digging duration: F(1,52) = 0.183, p-adj = 0.67; No. Burrows: F(1,52) = 0.00, *P*-adj = 1.00). To further investigate how BTB-induced activation of GPR65 in the knee joint affects the joint environment histological analyses were performed on the BTB-injected knees 24-h postinjection (Fig. 6*G*). While the thickness of the synovium did not differ between WT and KO animals (WT, 155.62 ± 23.13 µm, KO, 119.31 ± 14.27 µm, t = 1.34, df = 8.32, *P* = 0.22; Fig. 6*H*), a higher density of nuclei was observed in the synovium of WT animals (WT, 8.72 ± 0.39%, KO, 6.65 ± 0.72%, t = −2.57, df = 5.88, *P* < 0.05; Fig. 6*I*). Data here support the hypothesis that GPR65 mediates sensory neuron hyperexcitability, joint inflammation, and pain-like behaviors conferred by BTB administration and highlight the potential of GPR65 antagonists in the treatment of inflammatory conditions.

### Human FLS Express GPR65 and Arthritic Synovial Fluid Samples Activate GPR65.

**Fig. 7.**
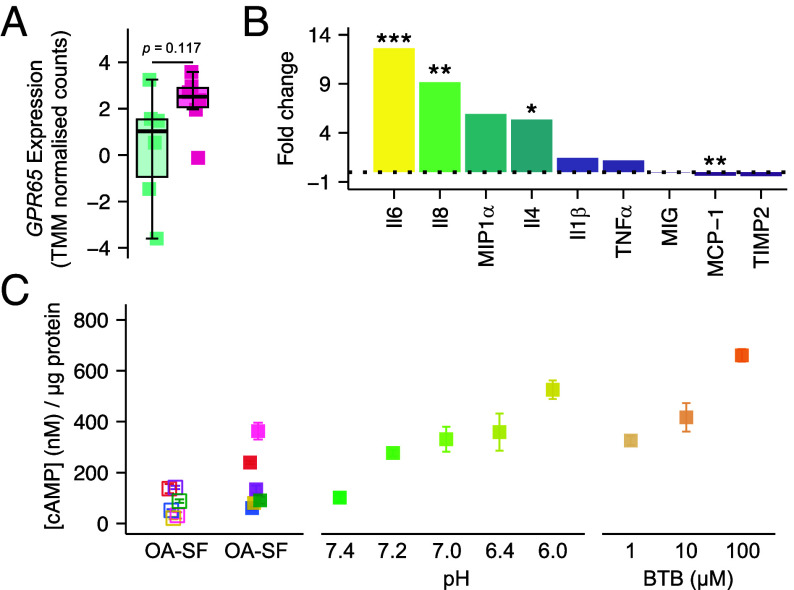
Human FLS express GPR65 and arthritic synovial fluid samples activate GPR65. (*A*) Expression of *GPR65* in synovium tissue from painful (pink) and nonpainful (green) sites of end-stage OA patients, data from Nanus et al. (51). (*B*) BTB-induced fold change in detection of inflammatory cytokines in conditioned media from stimulated human FLS. (*C*) Intracellular cAMP concentration of FlpIN CHO (open symbols) or mGPR65-CHO (closed symbols) cells, following stimulation with human OA synovial fluid samples, pH solutions, or BTB. *P-adj < 0.05, **P-adj < 0.01, ***P-adj < 0.001: (*A*) Published differentially expressed gene (DEG) data from Nanus et al. (51) was filtered to identify DEGs using two-group statistical comparison for >1.5 fold change and *P* < 0.05. (*B*) Two-way ANOVA followed by Bonferroni-corrected post hoc.

Having characterized a role of GPR65 in inflammatory joint pain in mice, the relevance of this signaling axis in human disease was explored next. Human OA patients can experience inflammatory flare ups, characterized by synovitis and increased experience of pain at inflamed sites (62). Examining RNA sequencing data from Nanus et al. (51), the transcriptomic profiles of OA patient synovium samples, biopsied from sites reported as painful and nonpainful, revealed that GPR65 displayed one of the greatest increases in expression at painful sites, compared to nonpainful sites of the proton-sensitive receptors, for end-stage arthritic patients (Fold change: 3.83, *P-adj* = 0.117; Fig. 7*A* and *SI Appendix*, Table S7), suggesting that GPR65 might contribute to increased experience of pain in OA. To further examine the potential role of GPR65 in the pathology of inflammatory arthritis in humans, FLS isolated from the painful sites of human OA patients were stimulated with BTB or DMSO for 24-h, after which media were collected and later assayed for cytokine levels. Compared to FLS exposed to DMSO, higher levels of inflammatory mediators were detected in the media of human FLS stimulated with BTB. Most notable increases included IL-6 (BTB, 0.740 ± 0.014, DMSO, 0.054 ± 0.011, *P-adj* = 0.0068; Fig. 7*B*), already a therapeutic target in arthritis (63) and IL-8 (BTB, 0.667 ± 0.001, DMSO, 0.066 ± 0.020, *P-adj* = 0.001; Fig. 7*B*), which is elevated in arthritic disease and has been shown to coordinate immune cell infiltration (64). Human FLS stimulated with BTB released less MCP-1, compared to cells incubated with the vehicle, DMSO (BTB, 1.635 ± 0.033, DMSO, 2.610 ± 0.004, *P-adj* = 0.0012; Fig. 7*B*), which contrasts with findings from mouse FLS, where MCP1 was among the most upregulated cytokines (Fig. 4*K*). Possible explanations for this may include a species-related difference on the reliance of certain mediators in inflammatory programs or an artifact of human FLS being isolated from OA patients, and so already influenced by the arthritic joint environment in addition to in vitro BTB stimulation, whereas FLS were obtained from healthy mice. Full analysis of the changes in cytokine levels following stimulation of human FLS are presented in *SI Appendix*, Table S4.

Experiments presented thus far have relied on activation of GPR65 by BTB, which although selective among PS-GPCRs, is a synthetic tool, not an endogenous mediator. To address this, and further establish whether GPR65 contributes to inflammatory joint pain, samples of synovial fluid collected from OA patients (*SI Appendix*, Table S9), were assessed for their ability to activate GPR65. The ability of samples to stimulate accumulation of cAMP in mGPR65-CHO cells was compared to responses seen in the parental Flp-IN CHO cell line (devoid of any endogenous proton-sensitive receptors). Of the six samples tested, three evoked a higher accumulation of cAMP in mGPR65-CHO cells than in parental cells, indicative of GPR65 activation (Fig. 7*C*). Alongside the synovial fluid samples, mGPR65-CHO cells were also challenged with acidic solutions or BTB, to compare the responses seen. Although the accumulation of cAMP observed cannot be equivocally pinned to engagement of GPR65 by protons in the synovial fluid, these data provide reassurance that the concentration of BTB selected for earlier studies was reasonable and still support a role of GPR65 in inflammatory arthritis pathology, suggesting further exploration as a therapeutic target is warranted.

## Discussion

Inflammatory conditions represent a significant burden on the quality of life for individuals affected, as well as having wider socioeconomic implications (3, 65, 66). Accordingly, there is a pressing need to develop better and safer medications to remedy both the inflammation and pain associated with chronic diseases, due to the limitations of currently used medications. Here, a role of the PS-GPCR GPR65 in the development of inflammatory joint pain is presented, offering insight into the cellular basis of inflammatory joint pain. The acidosis reported for inflammatory conditions, such as arthritis, makes a strong case for the involvement of proton-sensitive receptors in inflammation, however, delineating the roles of individual receptors is made difficult by the fact they are often coexpressed. To this end, the selective GPR65 agonist BTB (Fig. 1) was used to explore its role in inflammatory joint pain. Following injection of BTB into the mouse knee joint an inflammatory response is initiated, which is evident from joint swelling. Mice also demonstrate behaviors consistent with pain, including mechanical hypersensitivity of the afflicted joint 24-h after inflammation induction and longer lasting defects in digging behavior reminiscent of spontaneous pain and inflammation-induced apathy (Fig. 2). The finding that stimulation of sensory neurons with BTB alone could not recreate the hyperexcitability seen of neurons that directly innervate BTB-injected joints, suggested that another cell type may be involved (Fig. 3). FLS resident in the knee joint express higher levels of GPR65 than any other PS-GPCR, and stimulation of FLS with BTB resulted in accumulation of intracellular cAMP (Fig. 4*C*), activation of the transcription factor CREB (Fig. 5*H*) and release of proinflammatory cytokines (Fig. 4*G*). Incubation of neurons in conditioned media taken from BTB-stimulated FLS lead to neuronal hyperexcitability and greater voltage-gated inward currents, akin to the effects seen for knee-innervating neurons isolated from mice injected with BTB (Figs. 3 *F*–*H* and 4 *F*–*H*). It is thus postulated that the inflammation and associated pain resulting from intra-articular BTB injection at least partially arises from activation of FLS resident in the joint, which are triggered to release mediators that can stimulate immune cells and sensory neurons in the vicinity to drive inflammation and pain.

**Fig. 8.**
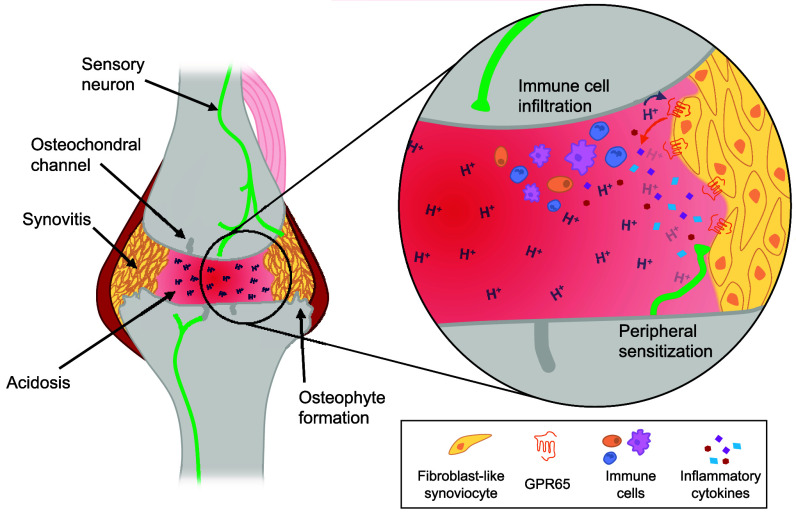
Activation of the proton-sensing GPCR, GPR65 on FLS contributes to inflammatory joint pain. Arthritic conditions are associated with localized acidosis of the joint environment and proliferation of FLS (synovitis). FLS express GPR65, are activated by the increased local concentration of protons (H^+^), leading to release of pro-inflammatory mediators capable of recruiting immune cells to the area, further driving inflammation. Mediators released by both FLS and immune cells act on sensory neurons that innervate the joint contributing to peripheral sensitization and the increased pain associated with arthritis.

Further evidence to support the specific involvement of GPR65 in inflammatory pain arise from the findings that GPR65 KO mice did not experience any inflammation or pain following injection of BTB (Fig. 6), FLS cultured from GPR65 KO mice did not exhibit BTB-induced cAMP accumulation (Fig. 5*A*) or release of mediators (Fig. 5*F*), and BTB-stimulated conditioned media from GPR65 KO FLS also failed to sensitize sensory neurons (Fig. 5*D*). These findings make use of the selective GPR65 agonist BTB, a convenient, but synthetic tool. The effect of protons, an endogenous GPR65 agonist, have also been explored throughout this study: BTB recapitulated most of the signaling effects of protons at GPR65 (Fig. 1) and protons were also shown to coordinate cAMP accumulation (Fig. 4*C*) and pCREB activation (Fig. 5*K*) for WT FLS, suggesting a similar mechanism may be initiated by natural acidosis which can occur during inflammatory arthritis. Furthermore, similar results were found for human FLS, and an ability of synovial fluid obtained from human OA patients to activate GPR65 was demonstrated. Integrating the findings reported here a potential mechanism for GPR65 in the mediation of arthritic pain may be postulated, whereby the acidosis of inflammation activates GPR65 expressed by FLS in the joint, which results in the production and secretion of inflammatory mediators which function to further recruit more immune cells, with mediators released by both FLS and the recruited immune cells capable of sensitizing nearby sensory neurons to coordinate heightened nociception (Fig. 8).

Stimulation of naïve cultures of sensory neurons with BTB alone was shown to be insufficient to recreate the hyperexcitability seen for neurons that project to BTB-injected joints (Fig. 3*F*). This might be explained by the very low expression of GPR65 by sensory neurons (67) and thus the involvement of other cell types was explored. Data reported here suggest a major role for FLS expressing GPR65 in establishing peripheral sensitization of sensory neurons when these cells are studied in isolation (Figs. 4 and 5). Activation of GPR65 through injection of BTB into the knee joint also coordinated joint inflammation, an increased density of nuclei among the synovium (Fig. 6*I*) and an increase in pain-like behaviors (Figs. 2 and 7). However, the KO mice used to attribute the inflammation and pain induced by BTB to GPR65 activation are global KO and it is thus impossible to resolve the contributions of individual cell types to the pathological changes observed. The involvement of other cell types cannot be overlooked, especially when immune cells, including T-cells and macrophages are also reported to express GPR65 (23). Given that most of the cytokines found to increase in concentration following BTB-stimulation of FLS are principally associated with immune cell recruitment, the BTB-stimulated immune cell infiltration and secondary wave of mediator release that will likely ensue, and the dependency of this on GPR65, is deserving of further investigation. Indeed, studying the identity of the increased number of cells present in the synovium of mice following injection of BTB, and subsequent activation of GPR65, and roles of these in observed pain responses remain to be studied. This might be addressed using more sophisticated tools for ablating GPR65 expression in specific cell types. Such an approach could add valuable cellular context to GPR65 activation, given there are reports of extracellular acidification reducing the ability of macrophages to produce TNF-α and IL-6 in a GPR65/PKA-dependent manner (68).

The principle means by which BTB and GPR65 have been linked to inflammatory pain in the present study is through the release of inflammatory mediators. Additional disease context has been added from studies of FLS and synovial fluid samples obtained from human OA patients. However, OA is primarily considered a degenerative disorder, rather than outright inflammatory condition, which may offer some explanation to the apparent inability of three of the OA synovial fluid samples to engage GPR65, which may have been obtained from patients experiencing less inflammation at the time of collection. Unfortunately, synovial fluid volumes available in this study prevented measurement of their pH, but wide variation of the pH of synovial fluid samples obtained from patients with various forms of arthritis has been reported (38–41). Accordingly, the next step should be to look at the involvement of GPR65 in conditions with a higher degree of inflammation, which might also enable an assessment of how the endogenous acidosis associated with such conditions contributes to pathology, a starting point could be the antigen-induced arthritis model, which has been shown to cause joint acidosis in rats (4) and might offer a more translational assessment of the therapeutic potential of targeting GPR65.

Considering that chronic inflammatory conditions, would result in prolonged periods of acidosis and findings reported here which indicate that GPR65 internalizes in response to acidic challenge (Fig. 1*G*), the contributions of GPR65 signaling from endosomal platforms to coordination of inflammatory pain is also worthy of further investigation, especially given the relevance of subcellular location on the ability of other GPCRs associated with inflammatory pain to coordinate nociception (69–71). This phenomenon represents an extra layer of intrigue for GPR65, and indeed other PS-GCPRs, due to the progressive acidification of the endomembrane system, which would likely impact upon receptor activity. The work presented here in addition to these discussion points, suggest that development of GPR65 antagonists may hold therapeutic value in the treatment of inflammatory pain, the nature by which the receptor would be activated and contribute to disease in a pathological setting, i.e. localized acidosis, also means that delivery of any future agents active against the receptor may be enhanced through exploiting recent developments in pH-sensitive drug delivery (72).

In summary, work described here has demonstrated that the PS-GPCR GRP65 is a regulator of inflammatory pain. The findings presented suggest that GPR65 coordinates inflammatory joint pain through FLS cells, which release inflammatory mediators in response to the GPR65 agonist BTB. These results provoke further questions regarding GPR65-driven inflammation and nociception, to allow better assessment of the receptor as a potential target for the development of anti-inflammatory and analgesic agents. Moreover, work here highlights the importance and potential value in further investigation of the wider PS-GPCR family in physiology and disease.

## Materials and Methods

Please see *SI Appendix* for comprehensive descriptions of methods.

### Ethical Approval.

Ethical approval for the collection and investigation of human-derived cells and synovial fluid samples was granted by the UK National Research Ethics Committee (14/ES/1044 and REC 16/SS/0172). Consent was provided by all patients. All animal work was regulated in accordance with the United Kingdom Animal (Scientific Procedures) Act 1986 Amendment Regulations 2012 and was approved by the University of Cambridge Animal Welfare Ethical Review Body.

### Animals.

Wildtype C57BL/6J mice (Envigo) and GPR65 KO mice (56) were housed in groups of up to five per cage with enrichments and ad libitum access to food and water. The holding room was maintained at 21 °C and operated a 12-h light/dark cycle. Mice were used at age 10 to 12 wk.

### In Vivo Studies.

Intra-articular injections, performed under anesthesia, were made through the patella tendon. Fast Blue (Polysciences) was used to label knee-innervating neurons. CFA was injected to model inflammatory arthritis. MIA was injected to model OA. BTB was injected to stimulate GPR65. DMSO served as a vehicle control for BTB injections. Knee inflammation was measured using digital calipers. Mechanical sensitivity of the knee joint was assessed using pressure application measurement (Ugo Basile) and digging behavior was assessed as an ethological readout of pain and well-being (3). Rotarod performance (Ugo Basile) was used to monitor mouse motor coordination. Behavioral analyses were carried out after blinding of experimental condition.

### Cell Culture.

Flp-In-CHO cells (Thermo Fisher) were used to generate stable PS-GPCR cell-lines. PS-GPCRs were cloned from mouse cDNA libraries using conventional restriction cloning methods. Expression of constructs by stable cells was confirmed through immunostaining and RT-PCR. Parental Flp-In-CHO cells were used as a control due to the lack of endogenous proton-sensitive receptor expression, or when experiments relied on transient transfection of tagged receptor constructs, i.e. bioluminescent resonance energy transfer (BRET) studies.

### Isolation and Culture of Mouse DRG Neurons.

Following humane killing of mice, lumbar DRG (L2-L5) were isolated and incubated in collagenase (3mg, 15 min; Merck) before incubation in trypsin (3mg, 30 min; Merck). Mechanical trituration was used to dissociate neurons, which were then plated on poly-D-lysine and laminin glass-bottomed dishes (MaTek). Where DRG were taken from mice injected with BTB, neurons from the ipsilateral and contralateral sides were collected and cultured separately. Overnight incubation of DRG neurons with BTB or DMSO was to a total volume of 2 mL, Stimulation of naïve neurons with BTB or DMSO was to a total volume of 2 mL, and 300 µL for FLS conditioned media.

### Isolation and Culture of Mouse FLS.

As conducted previously (50), mice patellae were exposed by resection of the quadriceps muscle and briefly washed in PBS before transfer to culture media. After a week of outgrowth patellae were discarded and cells passaged, FLS were enriched through three subsequent passages before experimentation.

### Isolation and Culture of Human FLS.

FLS were isolated from joint synovial tissues collected perioperatively from consenting OA patients following elective total joint replacement. Synovial tissue was prepared as previously described (73) in complete fibroblast media, and isolated FLS were enriched and maintained till 70% confluent up to passage four before plating for experiments.

### Cell Line Signaling Assays.

cAMP accumulation and ERK phosphorylation were quantified using LANCE Ultra TR-FRET reagents (Perkin Elmer). The Ca^2+^-sensitive dye, Fluo-4 (Thermo Fisher) was used to study Ca^2+^ mobilization. β-arrestin recruitment and receptor internalization were assessed by bystander BRET assays. Stock concentrations of BTB (prepared in DMSO) and psychosine [prepared in chloroform:methanol (1:1)] were diluted in Hanks Balanced Salt Solution, pH 7.4.

### cAMP Accumulation in FLS.

FLS were stimulated for 15 min followed by cell lysis. To account for the variability in cell number in experiments, cAMP concentration in lysates (inferred from a cAMP standard curve run in parallel) were normalized to the total protein concentration in each lysate (assessed by Bradford assay).

### qPCR.

RNA was isolated from FLS using TRIzol (Merck) and an RNA clean up and concentrator kit (Zymo Research). High-capacity reverse transcription reagents (Applied Biosystems) were used to synthesis cDNA and gene expression was assayed using TaqMan probes (Thermo Fisher). Relative expression was calculated as 2-ΔCt, where ΔCt is the difference in the Ct value obtained for the gene of interest minus that of the housekeeping gene, 18SrRNA.

### Electrophysiology.

Step-wise depolarization (Δ10 pA, 50 ms) was used to determine the rheobase. The activity of macroscopic voltage-gated channels was also assessed in voltage-clamp mode with appropriate series resistance compensation. Neurons were held at −120 mV for 150 ms before stepping to the test potential (−60 mV – 55 mV in 5 mV increments) for 40 ms and returning to a holding potential of −60 mV for 200 ms between sweeps. Peak inward and outward currents were normalized to cell capacitance. Fast Blue, knee-innervating, neurons were identified by LED excitation at 365 nm (Cairn Research).

### Cytokine Analyses.

FLS were stimulated with 100 µM BTB or 0.1% (v/v) DMSO in serum-depleted media for 24-h after which conditioned media were collected. Cytokine levels in collected media were assayed with Mouse or Human Inflammatory Antibody Arrays (Abcam) as appropriate.

### Immunostaining.

FLS were fixed by exposure to ice-cold 100% methanol and stained for CDH-11 and phospho-CREB. The nucleus was stained with DAPI. Cells were imaged with a Leica SP5 laser-scanning confocal microscope and 63x oil objective. The intensity of pCREB staining was analyzed in ImageJ.

### Histology.

BTB-injected knee joints were collected from WT and GPR65 KO mice 24-h after injections. Knees were fixed in 4% (w/v) paraformaldehyde and decalcified for 3 wk. 10 µm sections in the sagittal plane were collected using a cryostat. Sections were analyzed in ImageJ: the synovium thickness and nuclei density in the synovium were measured.

### Data Analysis and Statistics.

Data are presented as mean ± SE of the mean, the number of biological and technical replicates are detailed in individual figure legends. Appropriate analyses were selected according to the number of factors being compared and whether data met the assumptions for parametric analyses, the statistical tests employed are stated in corresponding figure legends. All analyses were performed in R, and *P* values < 0.05 considered significant.

## Supplementary Material

Appendix 01 (PDF)

## Data Availability

Datasets supporting the conclusions of this article are available in University of Cambridge Apollo Repository (74).
